# Prevalence and Clinical Features of Atopic Dermatitis in China

**DOI:** 10.1155/2016/2568301

**Published:** 2016-11-13

**Authors:** Xin Wang, Lin-Feng Li, Da-yu Zhao, Yi-wei Shen

**Affiliations:** ^1^Department of Dermatology, Beijing Friendship Hospital, Capital Medical University, Beijing 100050, China; ^2^Department of Dermatology, Beijing Shijitan Hospital, Capital Medical University, Beijing 100038, China

## Abstract

*Background*. The epidemiology of atopic dermatitis (AD) in Chinese outpatients is yet to be clarified.* Objectives*. To investigate population-based prevalence and clinical features of AD in Chinese outpatients.* Methods*. A multicenter cross-sectional study was conducted in outpatients with eczema or dermatitis from 39 tertiary hospitals in 15 provinces.* Results*. This study included 682 patients diagnosed with AD, with the mean age of 28.8 ± 20.1 years and the median course of 5.3 ± 6.9 years. AD patients had more severe itching (30.4% versus 13.8%, *p* < 0.001) and clinically suspected bacterial infection (21.7% versus 16.1%, *p* < 0.001) than those of other types of dermatitis. Older patients were more susceptible to have a history of flexion dermatitis (*p* < 0.001), bacterial infection (*p* = 0.005), and severe itching (*p* < 0.001). Outpatients with clinically suspected bacterial infection had 3.53-fold increased risk of AD than those without it (*p* < 0.001). The morbidity rate of AD in the (20–25°N) region is 2.86 times higher than that in the (40–45°N) region [OR (95% CI): 0.352 (0.241–0.514), *p* < 0.001].* Conclusions*. AD is characterized by unique clinical/demographic features. Bacterial infection and latitude region may have an impact on the incidence of AD in China.

## 1. Introduction

Atopic dermatitis (AD) is a type of dermatitis (inflammation of the skin), resulting in itchy, red, swollen, and cracked skin. As a common dermal condition, the incidence of AD is increasing annually worldwide including China. A recent systematic review of 69 cross-sectional and cohort studies has confirmed that AD is a worldwide phenomenon with a lifetime prevalence of >20% in many countries [1]. The prevalence of childhood AD is ranging from 15% to 30% and from 2% to 10% of adult AD in industrialized countries [2]. As we all know, many studies have shown that the prevalence of this disease among children has increased substantially over recent years [3]. In China, the prevalence of AD in students aged 6–20 years was only 0.7% in 2000 [4]; however, it had reached up to 8.3% (95% CI: 7.6%–9.1%) in children aged 3 to 6 years in Shanghai in 2012 [5]. Intriguingly, few studies have paid attention to AD patients of all ages, despite the fact that the occurrence of AD among adults has been shown to significantly affect socioeconomic conditions in both Asian and Western countries [6]. A recent survey has investigated clinical features of adult/adolescent (12 years and over) AD [7], but the epidemiology of AD at all ages in China is yet to be elucidated. Therefore, the purpose of the current study is to determine the actual prevalence and clinical features of AD at all ages in outpatients with dermatitis and eczema across large tertiary hospitals in China.

## 2. Methods

### 2.1. Study Population

This study has been approved by Institutional Review Board (IRB) committee at each hospital involved in this study. Oral informed consent was acquired from each participant before enrollment. All procedures were conducted according to the guidelines approved by the ethics committee at each hospital. Demographic and clinical information was acquired from patients who were treated in the respective hospitals from July 1 to September 30, 2014. Patients were diagnosed with AD in 39 tertiary hospitals of 15 provinces and municipalities in mainland China, including Guangdong, Chongqing, Hunan, Jiangxi, Henan, Zhejiang, Shanghai, Hubei, Jiangsu, Anhui, Shanxi, Beijing, Tianjin, Shandong, and Liaoning Province, that generally represented most regions of China.

### 2.2. Diagnostic Criteria of AD

All dermatologists involved in this study have abundant experience in clinical diagnosis and treatment for AD and have been trained in a standardized manner before the start of the project. First, each subject was inspected by a dermatologist independently. Then, a questionnaire survey was conducted by dermatologists after 10–15 minutes' dermatological physical examination. All diagnostic criteria mentioned in Williams diagnostic criteria [8] were integrated and recorded in detail; the parents or guardians had filled the consent forms for children patients. According to the questionnaire and physicians' evaluation, a comprehensive diagnosis of AD was made based on the gold standard “Williams diagnostic criteria” after investigation. Moreover, all patients with other types of dermatitis and eczema seen in the same period by the same investigators served as controls. Other types of dermatitis and eczema such as irritant contact dermatitis (ICD), widespread eczema, hand eczema, allergic contact dermatitis (ACD), neurodermatitis, seborrheic dermatitis, nummular eczema, asteatotic eczema, photo-contact dermatitis, autosensitization eczema, dyshidrotic eczema, and stasis dermatitis were classified based on International Classification of Diseases (ICD-10) [9] and diagnosed accordingly based on medical history and clinical features. No laboratorial test was performed for dermatologists to make the diagnosis.

### 2.3. Data Collection

All enrolled patients had completed a specific survey containing questionnaires regarding their general demographic characteristics, disease duration, severity of itching, lesion's distribution, type of skin lesion, and medical history. Itch was evaluated and divided into 4 levels: (i) no itching; (ii) mild itching that interrupted neither daily activities nor sleep of the participant; (iii) moderate itching that interrupted daily activities but not affected sleep; and (iv) severe itching that affected both daily activities and sleep of the participant. History of allergic disease, dry skin, infantile eczema, and flexion dermatitis was also recorded. Allergic diseases included asthma, allergic rhinitis, allergic conjunctivitis, and AD. Secondary bacterial infection was clinically suspected if superficial pustules, prudent exudation, or yellow colored crust was detected.

### 2.4. Statistical Analysis

Statistical analyses were processed by SPSS software (version 17.0). Differences in age and disease course between AD groups were analyzed by* t*-tests. Differences in age and disease course between AD among different age groups were analyzed by One-Way ANOVA. Differences in gender, all kinds of medical history, and clinically suspected bacterial infection between AD groups were analyzed by Chi-square tests. Itching grade between AD groups was analyzed by Chi-square test. Age, gender, and other significant factors were adjusted in Binary Logistic regression models for odds ratio (OR) and 95% confidence interval (CI) estimation. All analyses were two-sided with a significant level of *p* < 0.05.

## 3. Results

### 3.1. Demographic Characteristics of AD

Data were collected on a total of 8,758 outpatients with dermatitis and eczema. Overall, the prevalence of AD in outpatients was 7.8% (95% CI: 7.3–8.4). The comparison of demographic and clinical characteristics between AD and other types of dermatitis is summarized in Table 1. The mean age of AD patients was lower than those of other types of dermatitis (28.8 ± 20.1 versus 36.7 ± 18.4 years, *p* < 0.001), but median course was much longer (5.3 ± 6.9 versus 2.8 ± 4.8 years, *p* < 0.001). There is no difference in gender make-up (*p* = 0.071).

### 3.2. Clinical Characteristics of AD

78.4% AD patients had history of allergic disease, which was significantly higher than that of other types of dermatitis (9.3%, *p* < 0.001). Furthermore, history of dry skin, flexion dermatitis, and infantile eczema were more frequent than those of other types of dermatitis (73.6% versus 17.2%, 64.1% versus 5.5%, and 41.8% versus 6.6%, *p* < 0.001). In terms of the severity of itching, the AD patients may be more severe than that of other types of dermatitis patients (30.4% versus 13.8%, *p* < 0.001). The clinically suspected bacterial infection in AD patients was statistically significant higher than that of other types of dermatitis patients (21.7% versus 16.1%, *p* < 0.001).

**Figure 1:**
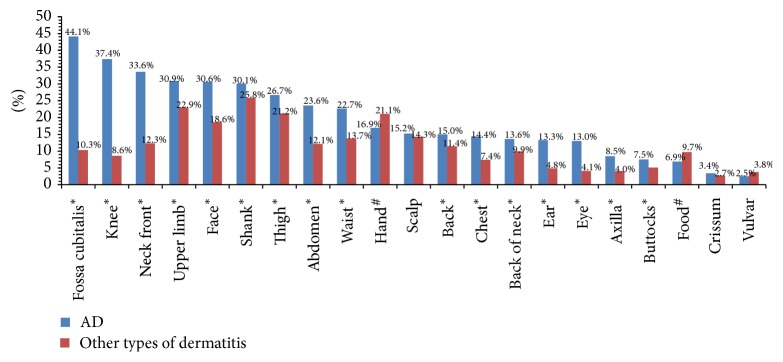
Lesions distribution of AD and other types of dermatitis. ^*∗*^This body location was more involved in AD than other types of dermatitis, *p* < 0.05, Chi-square test. ^#^This body location was more involved in other types of dermatitis than AD, *p* < 0.05, Chi-square test.

The top five frequent sites involved in AD were fossa cubitalis (44.1%), knee (37.4%), neck front (33.6%), upper limb (30.9%), and face (30.6%). The hand (21.1% versus 16.9%, *p* < 0.05) and foot (9.7% versus 6.9%, *p* < 0.05) were more frequent sites involved in other types of dermatitis than AD and little variation on scalp, crissum, and vulvar between them (14.3% versus 15.2%, 2.7% versus 3.4%, and 3.8% versus 2.5%, resp., *p* > 0.05); however, other body locations were much less involved in other types of dermatitis (Figure 1).

**Figure 2:**
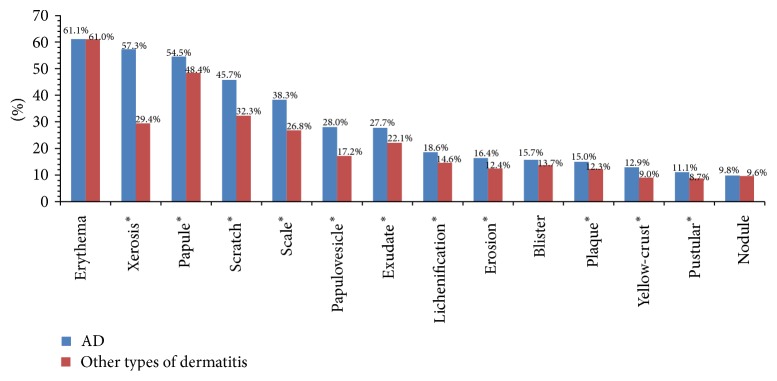
Skin lesion types of AD and other types of dermatitis. ^*∗*^This skin lesion type was more common in AD than other types of dermatitis, *p* < 0.05, Chi-square test.

The most common skin lesion types of AD were erythema (61.1%), xerosis (57.3%), papule (54.5%), scratch (45.7%), and scale (38.3%). All skin lesion types were more common in AD than other types of dermatitis, especially in xerosis, scratch, and scale (57.3% versus 29.4%, 45.7% versus 32.3%, and 38.3% versus 26.8%, resp., *p* < 0.05), but not in erythema, blister, and nodule (61.1% versus 61.0%, 15.7% versus 13.7%, and 9.8% versus 9.6%, *p* > 0.05) (Figure 2).

### 3.3. The Stratifying Analysis Based on Age Groups

Three stages of AD are proposed including infantile AD (age < 2 years), childhood AD (age 2–12), and adolescent/adult AD (age > 12 years). There were no significant statistical differences between three groups with respect to their sex (*p* = 0.58), history of allergic rhinitis (*p* = 0.87), dry skin (*p* = 0.78), and AD (*p* = 0.18). And there were high at both ends but low in the middle like a “U” shaped curve in the field of history of allergic disease (*p* = 0.005), asthma (*p* < 0.001), and allergic conjunctivitis (*p* = 0.024). As AD patients get older, they have higher proportion of history of flexion dermatitis (*p* < 0.001), clinically suspected bacterial infection (*p* = 0.005), and severe itching (*p* < 0.001), but lower proportion of history of infantile eczema (*p* < 0.001) (Table 2).

### 3.4. The Multivariable Logistic Regression Analysis of AD and Related Factors

In multivariable logistic regression analysis of AD and related factors, the independent indicators included age, disease duration, latitude, and bacterial infection.

It has indicated that the probability of developing AD increases 2.2% and 3.6%, respectively, as age and disease duration increased by one year. The outpatients with clinically suspected bacterial infection may have 3.532-times increased risk of AD than those without it. Thirty-nine hospitals were divided into 5 groups based on their latitude; the mean summer temperature increased as latitude decreased. The morbidity rate of AD in the lowest latitude region 20–25°N is 2.86 times higher than that in the highest latitude region 40–45°N (Table 3).

## 4. Discussion and Conclusion

AD is a global public health concern considering its great influence on people's life and social economy. There have been numerous epidemiological studies of AD using questionnaires, but very few have been performed by dermatologists' physical examinations owing to being time consuming and labor cost [10]. In order to guarantee uniformity of diagnosis, a panel of experienced dermatologists was involved in physical examination and detailed medical records. The present study has provided the first outpatient-based prevalence of AD at all ages in dermatology clinics in mainland China, with several interesting findings.

Our research shows that the incidence of AD in outpatients (7.8%) has been raised in recent years, much higher than that from our previous study (2.3%, 14/599) [11]. The higher prevalence and longer duration of AD compared with other types of dermatitis have indicated that more attention should be paid to this disease. Although the occurrence of AD may be associated with genetic factors [12, 13], a recent markedly increased incidence rate is more likely to be attributed to environmental factors [14–16] and changing lifestyles, such as air/water/soil pollution, an increase in aeroallergens, frequent bathing, and regular use of soap. In addition, the awareness of AD by dermatologists and patients may also contribute to this phenomenon. In China, the symmetrical eczematous dermatitis tends to be diagnosed as eczema in the past decades, indicating an overdiagnosed eczema and underdiagnosed AD [7]. Until recent years, we began to pay attention to AD and have improved the accuracy of diagnosis.

We have observed significant differences in terms of medical history between AD and other types of dermatitis. It has been widely accepted that AD is a systemic disease, not only dermal manifestations. AD in infancy is thought to contribute to the development of subsequent allergies known as atopic march [17]; more than half of children with moderate to severe AD would develop allergic rhinitis and/or asthma [18]. Up to 78.4% of AD patients had a history of allergic diseases, indicating that dermatologists should focus on medical history when a patient has symmetrical eczematous dermatitis. As AD patients were getting older, a "U" shaped curve was observed between age groups regarding a history of allergic diseases, asthma, and allergic conjunctivitis (Table 2). This phenomenon may confer the characteristics of allergic diseases. A study in Algeria has observed significant differences in the prevalence of asthma between different age groups, the highest in children aged <16 and in the oldest group (>54 years) [19]. Further investigation is required to explore mechanisms and significance of this phenomenon.

The proportion of AD patients with a history of flexion dermatitis or severe itching was positively associated with age (Table 2). Undoubtedly, scratching is one reason for this observation. This means a coexistent relationship between a history of flexion dermatitis and severe itching, which creates a vicious feedback loop of flexion dermatitis-severe itching-scratching.

Our study has indicated that AD is prone to clinically suspected bacterial infection, especially in older patients. A major cause for stratum corneum barrier disruption in AD is attributed to loss-of-function mutations in* FLG* gene that encodes filaggrin, a structural protein in keratinocytes/corneocytes, which allows outside-in penetration of foreign antigens and subsequent sensitization [20]. The AD patients with barrier-disrupted skin are more susceptible to bacterial infection, which in turn increases the severity of AD. In China, bacterial culture was not usually performed in clinics. This survey may have greater significance in clinical practice because how doctors define secondary bacterial infection would affect the choice of antibiotics.

Compared to other types of dermatitis, AD has more extensive body location involvement, especially in fossa cubitalis, knee, and neck front (44.1% versus 10.3%, 37.4% versus 8.6%, and 33.6% versus 12.3%, resp., Figure 1). The reason for this difference is unclear, probably related to thickness of cut-in and frequency of friction, for these parts of the body have thinner stratum corneum (SC) and are vulnerable to friction with the limbs activity. Conversely, the body locations with thick SC like hand and foot are not susceptible to suffer from AD, which is characterized by epidermal barrier dysfunction. AD has more diverse skin lesion types than other types of dermatitis, especially in xerosis, scratch, and scale (Figure 2). This has emphasized the importance of using emollients in AD patients, which is well tolerated and mainly in an attempt to restoring or replacing the intrinsic and/or externally induced abnormalities of the skin [21]. Those disparities of AD and other types of dermatitis are to some extent helpful to diagnosis in clinical practice.

Multiple factorial analysis has suggested a strong trend towards a decrease in the incidence of AD with an increase in latitude (Table 3), demonstrating dual roles of geography and economy. A large-scale prospective, longitudinal cohort study has evaluated the effect of long-term weather patterns on the severity of eczema symptoms in children and revealed that geographic areas with increased temperature, sun exposure (total, UVA, and UVB), and humidity were associated with poorly controlled disease [22]. It is possible that warm and humid weather leads to enhanced sweating, which has an irritant effect on the skin [23, 24]. The economic and living condition in southern China is becoming better than that in northern China, including more fastidious hygiene, better living conditions such as air conditioners, and higher standards of medical care. It is logistic to infer that economic condition may contribute to the higher rate of AD in southern China.

Several limitations should be considered when interpreting our results: (i) all of the 39 participating centers were tertiary referral hospitals located in 15 provincial capital or central cities, and most patients visiting these hospitals were in a better financial condition and medical insurance than the average people in China; (ii) the sample size in the present study was relatively small considering the total 1.3 billion Chinese population, and (iii) although we had demonstrated that clinically suspected secondary bacterial infection was quite reliable [25], bacterial culture is guaranteed to confirm the current findings. All these factors may lead to inevitable selection bias.

In conclusion, this research highlights atopic dermatitis with unique clinical characteristics, which has become a common dermatologic disease in China. Importantly, secondary bacterial infection and latitude region may have a profound impact on the incidence of AD.

## Figures and Tables

**Table 1:** Comparison of AD with other types of dermatitis.

Variables	AD (*N* = 682)	Other types of dermatitis (*N* = 8076)
Median age^*∗*^ (years, mean ± SD)	28.8 ± 20.1	36.7 ± 18.4
Median course^*∗*^ (years, mean ± SD)	5.3 ± 6.9	2.8 ± 4.8
Gender (men) (*n*, %)	368 (54.0)	4067 (50.4)
History of allergic disease^*∗∗*^ (*n*, %)	535 (78.4)	750 (9.3)
History of asthma^*∗∗*^ (*n*, %)	134 (19.6)	165 (2.0)
History of allergic rhinitis^*∗∗*^ (*n*, %)	184 (27.0)	289 (3.6)
History of allergic conjunctivitis^*∗∗*^ (*n*, %)	167 (24.5)	223 (2.8)
History of AD^*∗∗*^ (*n*, %)	87 (12.8)	100 (1.2)
History of dry skin^*∗∗*^ (*n*, %)	502 (73.6)	1388 (17.2)
History of flexion dermatitis^*∗∗*^ (*n*, %)	437 (64.1)	441 (5.5)
History of infantile eczema^*∗∗*^ (*n*, %)	285 (41.8)	534 (6.6)
Itching^*∗∗*^		
No (*n*, %)	0 (0.0)	259 (3.2)
Mild (*n*, %)	119 (17.4)	2510 (31.1)
Moderate (*n*, %)	356 (52.2)	4189 (51.9)
Severe (*n*, %)	207 (30.4)	1118 (13.8)
Clinically suspected bacterial infection^*∗∗*^ (*n*, %)	148 (21.7)	1302 (16.1)

^*∗*^ *p* < 0.001, *t*-test.

^*∗∗*^ *p* < 0.001, Chi-square test.

**Table 2:** Comparison of AD with different ages.

Variables	<2 years (*N* = 63)	2–12 years (*N* = 135)	>12 years (*N* = 484)
Median age^*∗*^ (years, mean ± SD)	1.3 ± 0.7	7.3 ± 3.1	38.4 ± 15.7
Median course^*∗*^ (years, mean ± SD)	0.7 ± 0.7	4.5 ± 3.5	6.1 ± 7.8
Gender (men) (*n*, %)	38 (60.3)	72 (53.3)	258 (53.3)
History of allergic disease^*∗∗*^ (*n*, %)	47 (74.6)	93 (68.9)	395 (81.6)
History of asthma^*∗∗*^ (*n*, %)	4 (6.3)	5 (3.7)	88 (8.2)
History of allergic rhinitis (*n*, %)	12 (19.0)	29 (21.5)	106 (21.9)
History of allergic conjunctivitis^*∗∗*^ (*n*, %)	11 (17.5)	15 (11.1)	104 (21.5)
History of AD (*n*, %)	8 (12.7)	11 (8.1)	31 (6.4)
History of dry skin (*n*, %)	44 (69.8)	100 (74.1)	358 (74)
History of flexion dermatitis^*∗∗*^ (*n*, %)	25 (39.7)	88 (65.2)	324 (66.9)
History of infantile eczema^*∗∗*^ (*n*, %)	60 (95.2)	117 (86.7)	108 (22.3)
Itching^*∗∗*^			
Mild (*n*, %)	10 (15.9)	15 (11.1)	94 (19.4)
Moderate (*n*, %)	44 (69.8)	85 (63)	227 (46.9)
Severe (*n*, %)	9 (14.3)	35 (25.9)	163 (33.7)
Clinically suspected bacterial infection^*∗∗*^ (*n*, %)	8 (12.7)	17 (12.6)	115 (23.8)

^*∗*^ *p* < 0.001, One-Way ANOVA test.

^*∗∗*^ *p* < 0.05, Chi-square test.

**Table 3:** Binary Logistic regression of AD and related factors.

	OR	95% CI	*p*
Age	1.022	1.009–1.035	0.001
Disease duration	1.036	1.018–1.053	<0.001
Gender			
Male	1.0 (ref.)		
Female	0.894	0.758–1.055	0.183
Latitude			
20–25°N	1.0 (ref.)		
25–30°N	0.733	0.511–1.051	0.091
30–35°N	0.821	0.637–1.059	0.129
35–40°N	0.858	0.655–1.122	0.263
40–45°N	0.352	0.241–0.514	<0.001
Clinically suspected bacterial infection			
No	1.0 (ref.)		
Yes	3.532	2.779–4.489	<0.001

Thirty-nine hospitals were divided into 5 groups based on their latitude: (i) 20°01′–25°N, Guangdong Province; (ii) 25°01′–30°N, Chongqing, Hunan, and Jiangxi Provinces; (iii) 30°01′–35°N, Henan, Zhejiang, Shanghai, Hubei, Jiangsu, Anhui, and Shanxi Provinces; (iv) 35°01′–40°, Beijing, Tianjin, and Shandong Province; and (v) 40°01′–45°N, Liaoning Province.
